# Towards sustainable antimicrobial therapies for *Staphylococcus aureus* skin infections

**DOI:** 10.1093/sumbio/qvae023

**Published:** 2024-09-02

**Authors:** Julia C Lang, Mariam Shahata, Keira Melican

**Affiliations:** AIMES-Center for the Advancement of Integrated Medical and Engineering Sciences, Karolinska Institutet and KTH Royal Institute of Technology, 17177 Stockholm, Sweden; Department of Neuroscience, Karolinska Institutet, 17177 Stockholm, Sweden; AIMES-Center for the Advancement of Integrated Medical and Engineering Sciences, Karolinska Institutet and KTH Royal Institute of Technology, 17177 Stockholm, Sweden; Department of Neuroscience, Karolinska Institutet, 17177 Stockholm, Sweden; AIMES-Center for the Advancement of Integrated Medical and Engineering Sciences, Karolinska Institutet and KTH Royal Institute of Technology, 17177 Stockholm, Sweden; Department of Neuroscience, Karolinska Institutet, 17177 Stockholm, Sweden

**Keywords:** bacterial pathogenesis, *Staphylococcus*, resistance, microbiome, medical microbiology

## Abstract

Skin and soft tissue infections (SSTIs) are a major economic and clinical burden. With the emergence of increasing antimicrobial resistance, novel treatment options, as well as advanced drug delivery systems will be essential to fight these infections and meet the UN Sustainability Development Goals (SDGs). SSTIs are commonly caused by *Staphylococcus aureus*, including the infamous MRSA (methicillin-resistant *S. aureus*). In this short review, we discuss new antimicrobial therapies with potential to combat skin infections caused by *S. aureus*. This includes discussion of antimicrobial strategies originating from both the host and microbiota. Adapting immunotherapy-type approaches to infection is also discussed, giving examples of cellular targets of interest. We examine the difficulties of therapeutic delivery into a barrier tissue such as skin and discuss exciting new developments in interdisciplinary approaches that may help overcome these challenges.

**Sustainability Statement**

SSTIs are a huge economic and clinical burden, and they diminish the health status of patients worldwide. Finding novel alternatives to antibiotics is in line with the United Nations Sustainable Development Goals to improve health and wellbeing (Goal 3). The heavy use of antibiotics in health care and in agriculture contributes to antimicrobial resistance that threatens health and is also an environmental burden for land, agriculture, and water. In this way, the developments described herein also address Goals 6, 14, and 15. In the later sections of this review, we discuss the use of novel materials for delivery and of antimicrobials, including microneedle and microspun fiber patches. It will be important to integrate sustainably sourced and manufactured materials into these approaches, which will be more effectively addressed now in the development stage rather than post-fact. As we develop new approaches to treat infection, it is important that sustainability is kept in focus in terms of health, environment, and material choices.

## Introduction

Skin and soft tissue infections (SSTIs) encompass a wide range of infections, from common, uncomplicated superficial boils and impetigo to complicated, life-threatening infections such as necrotizing fasciitis (Esposito et al. 2016, Linz et al. 2023). The variability of these infections relates to a number of factors, including the etiological pathogen, the rate of disease progression, severity, and anatomical location (Esposito et al. 2017). The most common bacterial cause of SSTIs is *Staphylococcus aureus* and recurrent *S. aureus* SSTI affects 16–19% of healthy adults (Vella et al. 2021). The increasing prevalence of community-acquired methicillin-resistant *S. aureus* (CA-MRSA) has been associated with a global rise in antimicrobial resistance (AMR) (Hersh et al. 2008, Pallin et al. 2008, Edelsberg et al. 2009, Frei et al. 2010). In 2022, The Lancet published a global estimation of bacterial AMR burden and identified *S. aureus* as one of six leading pathogens for AMR-associated deaths, and the leading cause in developed countries (Murray et al. 2022). The World Health Organization has listed methicillin-resistant *S. aureus* (MRSA) as a “priority pathogen” for R&D (WHO 2017). This review will discuss how the **development of new prevention and treatment approaches** to skin infections caused by *S. aureus*, is essential and will **contribute towards the UN Sustainable Development Goals, goal 3**, increasing good health and well-being. We also outline the development of novel antimicrobial therapy options for skin infection, with a focus on *S. aureus* and the challenges and advances of delivery of therapeutics into skin in a sustainable way.

## Skin environment and the microbiome

Skin is the largest organ of the human body, providing a protective interface to the surrounding environment (Gallo 2017). As part of the integumentary system, it provides both a **physical and an immunological barrier** (Scharschmidt and Fischbach 2013, Swaney and Kalan 2021). Similar to the gut, the skin is home to millions of fungi, viruses, and bacteria, building the extensive and site-specific **skin microbiota**, typically dominated by bacteria (Oh et al. 2014, 2016, Byrd et al. 2018). As the skin is a nutrient-poor and somewhat hostile environment (Scharschmidt and Fischbach 2013), coevolution of the commensal bacterial communities with their host provides mutual benefits (Kong and Segre 2012). The microbiota plays a direct role in the skin protective barrier with their ability to limit the introduction and expansion of exogenous microorganisms. This **colonization resistance** is facilitated by competition for the same niche and nutrients (Waaij et al. 1971, Buffie and Pamer 2013, Belkaid and Hand 2014, Swaney and Kalan 2021). The composition of the bacterial microbiota differs depending on the body site and can also change throughout life (Pistiner et al. 2008, Thavagnanam et al. 2008, Murk et al. 2011, Hooper et al. 2012, Sevelsted et al. 2015). The physiology of body sites can be characterized as moist, dry, or sebaceous, with factors such as pH, temperature, moisture, sebum content, and topography that vary at different sites (Grice et al. 2009, Grice and Segre 2011, Byrd et al. 2018). Across most human skin locations, the **staphylococcal species** are a prominent part of the microbiota (Byrd et al. 2018, Joglekar et al. 2023, Severn and Horswill 2023). The most common species is *S. epidermidis*, a ubiquitous coagulase-negative Staphylococci (CoNS) comprising >90% of the skin resident microbiota (Cogen et al. 2008, Paranthaman et al. 2023). Within the human population ∼20% are also persistently colonized with the potentially pathogenic, coagulase-positive *S. aureus*, and another 30% are transient carriers (Otto 2010a). Carriage rates can vary with geographic location (Sollid et al. 2014), age (Lebon et al. 2008, 2010, Olsen et al. 2013), and sex (Graham et al. 2006, Zanger et al. 2012, Sollid et al. 2014). The anterior nares are recognized as the major colonization site (Kuehnert et al. 2006, Brown et al. 2014). While being colonized with *S. aureus* is a risk factor for invasive infections, including **skin and soft tissue infection (SSTIs)** or bacteremia, colonized patients paradoxically show less mortality (von Eiff et al. 2001, Wertheim et al. 2004). The presence of antibodies against *S. aureus* indicates that almost all of us are colonized, usually asymptomatically, at some point (O'Gara 2017).

The skin microbiota also plays an important role in the **education of our immune system** (Naik et al. 2012, Belkaid and Segre 2014, Xing and Naik 2019). This education is believed to occur within a critical time window in the neonatal period and early life with long-lasting impacts on immune homeostasis and susceptibility to infections and inflammatory diseases (Torow and Hornef 2017, Reynolds and Bettini 2023). Caesarean section and the use of antibiotics in infants are examples of perturbating factors that have been associated with a disrupted early life microbiota resulting in an increased risk for asthma, allergy, and atopic dermatitis (AD) (Pistiner et al. 2008, Thavagnanam et al. 2008, Murk et al. 2011, Sevelsted et al. 2015). The important role of the microbiota on immune development has been shown in mice that are raised in germ-free environments and exhibit significant differences in the cellular composition of the immune system (Hooper et al. 2012). Restoring the microbiota by bacterial colonization after the weaning period only partially restored the immune development, demonstrating the time frame for building life-long immune homeostasis (Hooper et al. 2012). Early colonization by *S. epidermidis* has been shown to result in highly activated regulatory T cells (Tregs) accumulating in the skin (Scharschmidt et al. 2015, 2017). The interaction between a subset of skin resident dendritic cells with colonizing *S. epidermidis* induces skin homing of CD8 + T cells that contribute to skin inflammation and defense (Naik et al. 2012, 2015). The recognition of *S. epidermidis* lipoteichoic acid via TLR2, an innate immune receptor on sentinel cells, can inhibit cytokine release and thereby suppress skin inflammation and promote wound healing (Lai et al. 2009). Besides playing an important role in controlling T effector and regulatory T cell equilibrium, CoNS can regulate the expression of complement genes in skin, whereas disruption of complement signals alters the composition and diversity of the skin microbiota (Chehoud et al. 2013). Skin-resident commensals, including *S. epidermidis*, can also induce skin cells to express antimicrobial peptides (Brandwein et al. 2017). These findings highlight the crosstalk between colonizing skin microbiota and the host tissue, which modulates niche-specific immunity and protection. How the skin develops selective tolerance towards surface commensals and differentiates them from potential pathogens is, however, not yet fully understood (Xing and Naik 2019). Miseducation resulting in immune tolerance to pathogens would increase the infection susceptibility of the host, while failure in tolerance to commensals could result in aberrant immune reactivity to resident microbiota. Early life microbiota exposure is thus important for maturation of protective immunity but also for establishing tolerance towards commensal colonizers.

## Skin disease/infections

A dysbiosis of the skin microbiota or skin barrier disruption is associated with the development of skin and soft tissue infections (Lee and Kim 2022, Smythe and Wilkinson 2023) as well as skin diseases such as atopic dermatitis (Kong et al. 2012, Sanford and Gallo 2013) and psoriasis (Stehlikova et al. 2019) (Table 1). Host genetic predispositions and increased microbial density, or microbial dysbiosis, together with an impaired skin barrier can result in a locally increased immune response as well as inflammation that contributes to pathology (Belkaid and Segre 2014). *Staphylococcus aureus* is the most common cause of SSTIs (Eron et al. 2003, Vinh and Embil 2005, Dryden 2009, Jeng et al. 2010, Ray et al. 2013) and enriched in atopic dermatitis lesions (Blicharz et al. 2019, Conte et al. 2023) that commonly become superficially infected (Alexander et al. 2020).

**Table 1.** Common bacterial skin and soft tissue diseases and the skin layer most affected.

Disease	Organism	Affected skin layer	Reference
Cellulitis	*S. aureus, S. pyogenes*	Dermis and subcutaneous tissue	Swartz Morton (2004)
Staphylococcal scalded skin syndrome	*S. aureus*	Epidermis	Ladhani and Evans (1998)
Impetigo	*S. aureus, S. pyogenes*	Epidermis	Hartman-Adams et al. (2014)
Folliculitis	*S. aureus*	Epidermis and hair follicle	Luelmo-Aguilar and Santandreu (2004)
Atopic dermatitis	*S. aureus*	Stratum corneum and Epidermis	Blicharz et al. (2019), Conte et al. (2023)
Psoriasis	*S. aureus, S. pyogenes*	Epidermis	Fry and Baker (2007)

Work from our own lab has shown that colonization of the surface of human skin alone is not enough to cause invasive infection (Schulz et al. 2019). Barrier breach has long been speculated as a key step in skin and soft tissue infections, which allows exogenous or colonizing bacteria to enter deeper tissue (Wanke et al. 2013). Exciting recent work has indicated that colonization of *S. aureus* on skin may in and of itself cause a nerve-driven itch response, leading to minor disruptions of the skin barrier and subsequently higher risks of skin infection (Deng et al. 2023). This cross talk between bacterial colonization and itch is of particular relevance in skin disorders such as atopic dermatitis, which are characterized by itch and barrier dysfunction (Gallo and Horswill 2024). More obvious skin barrier breaches include wounds and injuries but also surgical interventions and indwelling medical devices (O'Gara 2017). In the hospital setting, indwelling catheters, such as venous lines, are at high risk of becoming colonized with *S. aureus* and other skin commensals. The abiotic surface of the catheters appears to be a particularly good adherence surface for these bacteria, from which they can form biofilms, bacterial communities embedded in a complex extracellular matrix (Brandwein et al. 2016). Biofilm formation complicates treatment and clearance of a number of infections but is particularly problematic in indwelling device infections and chronic wounds (Zhao et al. 2013). Bacterial colonization and biofilm formation in venous lines can lead to bloodstream infections and often require replacement, increasing treatment times and costs (Esposito et al. 2013, Nakamura et al. 2015). Biofilm formation also complicates infections within wound environments making it difficult for pharmaceuticals to penetrate the bacterial biofilm or allow immune cells to clear the bacteria. The biofilm matrix structure appears to protect the bacteria from desiccation to physical removal in the complex environment (Zhao et al. 2013, Kruse et al. 2015). Chronic wounds are often polymicrobial, with *S. aureus* and *Enterococcus faecalis* common co-colonizers. The study of polymicrobial infections has revealed an extensive cross-talk between the different pathogens, which is helping shape our understanding of how to best design new treatments for these types of infections (Ch’ng et al. 2022, Kao et al. 2023).

The current standard care for SSTI infections is **antibiotic treatment** (Silverberg 2021, Sartelli et al. 2022). Antibiotics may be applied either topically or systemically dependent on the severity of the infection. Topical antibiotics, which are often used in the case of minor infections such as impetigo include mupirocin, fusidic acid, bacitracin, and polymyxin (Bandyopadhyay 2021). Topical antibiotics are also used for decolonization, where the aim is to eliminate colonizing MRSA on the skin or in the nares of carriers (Kao and Fritz 2024). Advantages of topical treatment include accessibility to the site as well as less impact on other microbial flora. Disadvantages include a risk of skin irritation, difficulty in penetration, and the development of resistance (Bandyopadhyay 2021). While there is certainly a place for topical antibiotic treatment, effectivity and reliability could be improved. A number of clinical trials have indicated that systemic delivery of antibiotics leads to better cure rates and lower incidence of recurrence of skin infections (as reviewed in Kao and Fritz 2024). Systemic antibiotic delivery requires high dosages to achieve necessary concentrations in the target tissue, which results in high serum levels and potential ototoxicity and nephrotoxicity (Schentag et al. 1978, Wahlig et al. 1978, DeCoster and Bozorgnia 2008). The use of systemic broad-spectrum antibiotics can perturb the beneficial microbiota alongside the intended effect on the pathogens (Josefsdottir et al. 2017, de Nies et al. 2022, Patangia et al. 2022, Smythe and Wilkinson 2023). The reduction of microbiome diversity and dysbiosis can lead to development of long-term conditions that impact human health as described earlier and the spread of antibiotic-resistant bacteria (Patangia et al. 2022). As multidrug resistance is rising, and the significant side effects of systemic drug administration become apparent, the **search for alternative treatment strategies in skin diseases and infections** has increased.

## Current research frontier for antimicrobial therapies for skin infections

**Figure 1.**
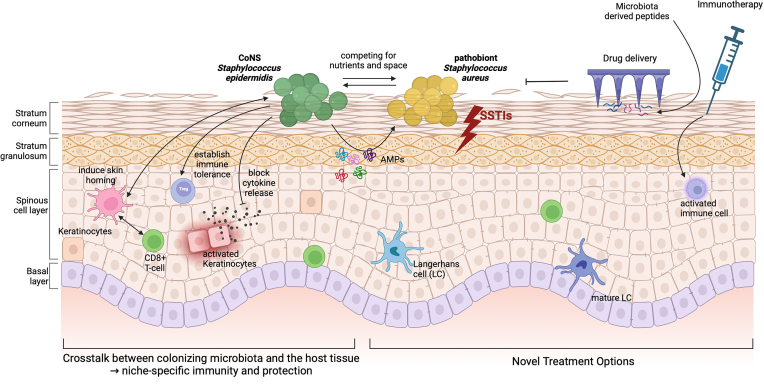
Schematic overview illustrating discussed elements of skin-specific immunity, SSTIs caused by *S. aureus*, and potential pathways and delivery mechanisms for novel treatments. It also depicts the interaction between skin-colonizing CoNS, such as *S. epidermidis* and *S. aureus*. The annotated schematic refers to different sections of this review. Created with BioRender.

These challenges described above lead to the exploration of alternative and complimentary treatment strategies. Treating SSTIs effectively, at the early stages, will contribute to better health and more sustainable clinical care. Effective local treatment and prevention techniques will help ease the medical and environmental burden of antibiotic use. This research is already well underway and in the following sections we will outline some of the most promising emerging technologies. We will discuss approaches from two broad perspectives: strategies that focus on the host and those from the microbial domain. Of particular interest is the integration of interdisciplinary research, which brings together basic microbiological research with advanced materials and nanotechnologies to find new solutions for more sustainable treatment and prevention strategies. Figure 1 graphically summarizes the approaches discussed.

### Host-mediated strategies

A conundrum for those studying SSTIs caused by Staphylococci is the fact that *S. aureus* harmlessly colonizes up to 30% of the population (Otto 2010a). This means that in the vast majority of cases where human skin is exposed to *S. aureus*, the host immunity controls this potential pathogen, and no clinical infection occurs. Harnessing this immune response is a promising pathway for new treatments. Immunotherapy is not a completely new concept for infection. Vaccination is a form of immunotherapy, triggering the adaptive immune response by exposure to microbial antigens. Extensive research has been performed into developing a vaccine against *S. aureus*, but the challenges are complex and well described in a more targeted review (Fowler and Proctor 2014). One noted complication in vaccine development is the lack of translatability of mouse trials into human immune protection (Otto 2010b). Immunological targets that have been used in active immunization trials include *S. aureus* virulence factors such as alpha-toxin, toxic shock syndrome toxin, components of the biofilm matrix, or iron-regulated proteins (Otto 2010b). Beside active immunization strategies, passive immunization that eliminates major virulence determinants in *S. aureus* was also considered. But thus far, we still do not have an effective vaccine for *S. aureus*.

Targeted and controlled forms of immunotherapy have shown incredible efficacy in other medical specialties, such as cancer treatment, where they interact with specific cell types and cell processes (Cappell and Kochenderfer 2023). Immunotherapy approaches, and particularly those that focus on the early innate immune response hold potential for infectious diseases, though they are only in the very early stages of development. The first step for SSTI is understanding the inherent immune protective systems of the human skin. Identifying how our skin can differentiate its beneficial microbiota from potentially pathogenic bacteria will help identify microbe-specific pathways that can be augmented by immunotherapy. A challenge of this approach for *S. aureus* is the plethora of immune evasion strategies this bacteria employs (Pier 2013, Rasigade 2018). The wide range of staphylococcal virulence factors with often functional redundancy means that targeting a single factor or immune pathway is unlikely to be sufficient. Blocking multiple pathways simultaneously, in a polyvalent approach may hold more promise (Otto 2010b). In terms of developing this immunotherapy, identifying which cells to target in the skin and understanding the pathways by which commensal bacteria modulate protective immunity are critical. Skin commensal bacteria have been shown to drive IL-1 signaling to modulate resident T lymphocyte function and induce protective immunity in mouse skin (Naik et al. 2012). Commensal-specific T cell responses were shown to result from the coordinated action of skin-resident dendritic cell subsets in response to colonization by *S. epidermidis* and were not associated with inflammation, rather with commensal-specific immune protection (Naik et al. 2015). Dermal dendritic cells have been identified as important skin sentinels, which can differentiate *S. aureus* from the commensal *S. epidermidis* by a pro-inflammatory and α-toxin-triggered IL-1β response (Leech et al. 2019, Xing and Naik 2019). Recent work in our lab has translated this work into a human skin model and shows an increased IL-1β response to colonizing *S. aureus* compared to *S. epidermidis* (Lang et al. 2024). How these skin-resident immune cells differ between bacterial friend and foe has been shown to depend upon the make-up of the bacteria cell wall, the wall-teichoic acid structure (van Dalen et al. 2019). An increasing focus on the interactions between the skin resident immune sentinels and bacteria is leading to the very real possibility that we can find ways to enhance or augment this protective mechanism to prevent skin infections, reminiscent of immunotherapies used in cancer therapy to target specific cell types and cell processes.

Skin tissue, like many other barrier tissues, also has an extensive chemical arsenal at its disposal. Antimicrobial peptides (AMPs) are small peptides produced by the skin that can directly mediate an antimicrobial effect. AMPs are among the first line defence against pathogens and are considered a key element in the innate immune system (Joshi et al. 2023). These peptides are produced by various cell types of the skin, such as keratinocytes, mast cells, and eccrine sweat glands. On the human skin, many AMPs have been identified in protecting the skin from pathogen infections. These AMPs include human β-defensins (hBD) 1–3, psoriasin (S100A7), and cathelicidin LL-37 (Bardan et al. 2004). Some AMPs are secreted constantly, maintaining a base level of protection; however, studies have shown that the production of AMPs increases upon microbial stimulation (Dinulos et al. 2003, Bardan et al. 2004, Nagy et al. 2006). AMPs have several mechanisms of action against pathogens, including targeting of the cell membrane of the pathogen. Most AMPs are cationic while the cell walls of gram-positive and gram-negative bacteria have an overall negative charge generating an electrostatic attraction, which initiates cell wall destruction (Luo and Song 2021). AMPs can inhibit biofilm formation with studies showing that LL-37 can disrupt the cell signaling system and reduce bacterial attachment to the skin (Overhage et al. 2008). AMPs have long held promise as an alternative treatment modality and there are exciting developments in delivery and testing currently ongoing (Patrulea et al. 2020).

### Microbiota-derived therapy

During the long co-evolution of bacteria on human skin, the different members of the skin microbiome have developed their own antimicrobial systems. Even within the *Staphylococcus* genus, there is significant cross-talk. Notable commensal coagulase negative *Staphylococcus* on the skin includes *S. lugdunensis, S. hominis*, and the omnipresent *S. epidermidis* (Grice and Segre 2011). Crosstalk between the skin microbiota gives the bacteria the ability to manipulate and modulate their local microbial environment as they compete for nutrients and space, but also provides an opportunity for us to exploit these inherent antimicrobial approaches. Microbial derived AMPs were discovered as early as 1925 and their mode of action has been extensively characterized with each exhibiting its own antimicrobial killing efficacy, immunomodulatory functions, and chemotactic activity (Kumar et al. 2018, Mookherjee et al. 2020). A subset of bacterial-derived AMPs are bacteriocins that target and kill or inhibit other microorganisms, often closely related species (Cavera et al. 2015, Benítez-Chao et al. 2021). An overview of staphylococcal bacteriocins can be found in Table 2. *Staphylococcus epidermidis*, e.g. produces a number of different bacteriocins and molecules that protect the skin from potential invaders, including Pep5, Epilancin K7, and Epidermin, which can be effective against other gram-positive bacteria, as well as other *S. epidermidis* strains that do not produce these bacteriocins (Christensen and Brüggemann 2014, O'Sullivan et al. 2019). Staphylococcal isolates from the nasal cavity, including *S. lugdunensis*, also show antimicrobial activity against *S. aureus*. Recently, a novel thiazolidine-containing cyclic peptide, lugdunin, produced by *S. lugdunensis* was identified, which inhibits *S. aureus* growth and epithelial colonization (Zipperer et al. 2016). Lugdunin increases the expression and release of LL-37, a cathelicidin-derived human AMP, and the murine chemokine CXCL8/MIP-2 in human keratinocytes and mouse skin, respectively (Bitschar et al. 2019). With its direct antimicrobial effect, its immunomodulatory functions on the innate immune response in skin, and the synergy with other microbiota-derived factors, lugdunin can provide protection against *S. aureus* on multiple levels (Bitschar et al. 2019). Lugdunin is the first reported non-ribosomally synthesized peptide from human microbiomes, building a new class of short peptide bacteriocins (SPBs) named fibupeptides. Other work has identified strain-specific bacteriocins produced by CoNS, including *S. epidermidis* and *S. hominis* that are highly potent and selectively kill *S. aureus* in synergy with host AMPs (Zipperer et al. 2016, Bitschar et al. 2019, Krauss et al. 2020, Joshi et al. 2023). These strains also produce short peptide bacteriocins (SPBs) called Epidermin (Götz et al. 2014) and Hominicin (Kim et al. 2010), respectively. To test the efficacy of this exciting class of commensal derived molecules, AMP producing CoNs were introduced into AD patients lacking commensal *S. epidermidis* and *S. hominis*, and this treatment significantly decreased colonization with *S. aureus* (Nakatsuji et al. 2017). While healthy human skin harbors several different bacterial species with anti-*S. aureus* activity, the atopic dermatitis patients in this study were lacking these AMP producing commensal strains. The specificity for *S. aureus* but not other commensals could make the use of bacterial AMPs a superior approach to promote cutaneous homeostasis. A successful re-colonization of antimicrobial commensals on the skin surface could result in long-term protection against *S. aureus* colonization but efficacy and potential side-effects need further evaluation. Synthetic copies of AMPs and antimicrobial molecules that are naturally used as bacterial interspecies competition may offer any opportunities for novel skin therapeutics and complement the treatment of skin diseases while preserving the microbiome as a balanced community. However, concerns about development or natural occurrence of AMP resistance in bacteria and their cross-resistance must be part of the discussion.

**Table 2.** Exemplary bacteriocins isolated from staphylococcal species.

Producer	Bacteriocin	Main mode of action	References
*S. aureus*	Aureocin	Dissipate membrane potential and stop biosynthesis of DNA, polysaccharides, and protein	Netz et al. (2001, 2002a, b)
*S. aureus*	C55	Pore formation in the cytoplasmic membrane	Navaratna et al. (1998)
*S. aureus*	Aureocyclicin	Putative pore formation	Potter et al. (2014)
*S. epidermidis*	Epidermin	Peptidoglycan biosynthesis inhibition	Bonelli et al. (2006), Fontana et al. (2006), Nascimento et al. (2006)
*S. epidermidis*	Epicidin	Putative pore formation	Heidrich et al. (1998)
*S. epidermidis*	Epilancin	Not well understood, potent in disrupting the membranes of liposomes composed of negatively charged membrane lipids	Ekkelenkamp et al. (2005), Wu et al. (2023)
*S. epidermidis*	Pep5	Autolysis of the target cell due to release and activation of cell wall hydrolyzing enzymes	Kaletta et al. (1989), Bastos et al. (2009)
*S. epidermidis*	Epidermicin	Disruption of bacterial membranes	Sandiford and Upton (2012), Halliwell et al. (2017), Hammond et al. (2020)
*S. hominis*	Hominicin		Kim et al. (2010)
*S. lugdunensis*	Lugdunin	Dissipation of a bacterial cell membranes	Zipperer et al. (2016), Ruppelt et al. (2024)
*S. hyicus*	Hyicin		Duarte et al. (2018)
*S. capitis*	Nisin	Pore formation in the membrane and inhibition of cell wall biosynthesis	Wiedemann et al. (2001), O'Sullivan et al. (2019)
	Capidermicin	Putative pore formation	Lynch et al. (2019)
*S. gallinarum*	Gallidermin	Inhibition of murein and wall teichoic acid biosynthesis	Saising et al. (2012), Götz et al. (2014)
*S. warneri*	Warnericin	Membrane destabilization by detergent-like mode of action	Minamikawa et al. (2005), Verdon et al. (2009)
*S. simulans*	Lysostaphin	Degradation of peptidoglycan	Schindler and Schuhardt (1964), Gründling et al. (2006)

In addition to antimicrobial substances, bacteria of the skin microbiome have other mechanisms of interspecies competition and interference. For *Staphylococcus*, the accessory gene regulatory (*agr*) quorum sensing (QS) system is a way in which bacterial populations can communicate and respond to their environment and status in their habitat (Geisinger et al. 2012, Canovas et al. 2016). Upon reaching a certain bacterial density, the levels of secreted auto-induced peptides (AIPs) increase, which can bind to the kinase receptor AgrC. This binding activates the response regulator (AgrA), which ultimately results in the production of virulent factors such as toxins. In simplified terms, Agr can be seen as a virulence switch, as activation of *agr* often leads to increased virulence. It has been shown that infants colonized by *S. aureus* that had dysfunctional Agr quorum-sensing were less likely to develop AD (Nakamura et al. 2020), demonstrating the importance of this system in skin diseases. Not only is a bacterial population affected by its own QS system, but competing bacterial populations can interfere with other strains QS systems via heterologous AIPs acting as competitive inhibitors (Geisinger et al. 2012, Williams et al. 2019). This quorum quenching mechanism is believed to result in growth inhibition or altered expression of virulence genes and can be advantageous to one bacterial species (Grandclément et al. 2016). When it comes to treatment strategies, quorum sensing inhibitors (QSIs) targeting different stages of the QS machinery have been suggested as anti-virulence compounds, which can modify the expression or activity of virulence factors rather than the viability (Naga et al. 2023). Due to their critical role in the pathway, AgrC and AgrA are the most common inhibition targets. A naturally occurring Agr inhibitor is Solonamide B. Solonamide B was shown to bind AgrC, preventing AIPs from binding. Other studies have also shown that by reducing Agr activity, Solonamide B can reduce *S. aureus* neutrophil killing (Nielsen et al. 2014). A combination of synthetic heterologous AIPs that reduce the proteolytic activity of *S. aureus* against bacteriocins, a defense/resistance strategy, together with efficient antimicrobial bacteriocins could further improve therapeutic outcome (Canovas et al. 2016). Quorum inhibition has however been met by some controversy and much work is still needed to validate it’s effectivity and potential side-effects *in vivo*, and particularly in clinically relevant model systems as has been reviewed elsewhere (Otto 2023).

Other microbiota-derived metabolites with inhibitory functions on the growth of opportunistic skin pathogens are short-chain fatty acids (SCFAs). They are mostly produced in low concentrations by the gut microbiota but also by skin commensal bacteria, such as *S. epidermidis* (Xiao et al. 2023). It has been shown that SCFAs influence inflammatory disease symptoms and mediate cytokine and chemokine production (Furusawa et al. 2013, Smith et al. 2013). For example, the SCFA butyrate, produced by *S. epidermidis* via glycerol fermentation, inhibited *S. aureus* growth and attenuated skin inflammation (Traisaeng et al. 2019). There is growing evidence for using SCFA butyrate as a therapeutic agent for treating inflammatory skin conditions in AD patients with *S. aureus* infection (Luo et al. 2023). It has also been shown that free fatty acids can be produced by lipolytic cleavage of lipids secreted from the sebaceous glands on skin and that they can control skin colonization (Fluhr et al. 2001, Drake et al. 2008). The main mechanism is destabilization of the phospholipid membranes of the bacteria, leading to pore formation and lysis with comparable potency to AMPs (Georgel et al. 2005, Desbois and Smith 2010, Yoon et al. 2018). The presence of fatty acids on the skin also regulates pH and can affect bacterial virulence gene expression (Desbois and Smith 2010, Yoon et al. 2018). Resistance development by various species to these mechanisms is, however, a noted problem (Clarke et al. 2007, Drake et al. 2008). Further research could help overcome these obstacles to exploit the promising use of fatty acids for antibacterial applications. The approaches we discuss here focus on microbial-derived antimicrobials. Probiotics and the addition of additional bacteria is another field of research within this domain. While extensively used in promoting gut health and the balance of gut microbiome, the use of topical probiotics in skin diseases has been less well established but holds some promise and is well described in more targeted reviews (Habeebuddin et al. 2022).

## Delivery of therapeutics

One of the greatest properties of human skin is its barrier function. This impressive barrier protects us from many insults but can become a problem when it comes to the delivery of treatments for SSTIs. As discussed above, systemic treatment is often not optimal for localized SSTIs due to both the lack of penetrance into the skin but also the systemic side-effects of antibiotic therapy on gut microbiome and other beneficial bacterial communities. An important aspect of ongoing research is into the mechanisms by which we can deliver potential new treatments to human skin.

Infected wounds pose a particular challenge for delivering antimicrobial agents. This includes achieving a sufficient and homogenous antimicrobial concentration, local pH variabilities that may change drug performance (Lamp et al. 1992, Emrich et al. 2010, Percival et al. 2014, Jones et al. 2015), inflammation, biofilms, altered bioavailability, and multi-species infections (Smith et al. 2020). However, these variables also offer opportunities for specific drug delivery systems, which react to different stimuli. A moist wound environment, e.g. is helpful in terms of healing and promotes a lower pH, which creates a more hostile environment for pathogens (Winter 1962, Hinman and Maibach 1963, Junker et al. 2013, Kruse et al. 2015, Jaiswal et al. 2016). Keeping a moist wound environment can, however, promote bacterial and fungal growth and can lead to tissue breakdown (Zeng et al. 2018). Bacterial biofilm formation is common in chronic wounds and these structures decreased antimicrobial efficacy due to the physical barrier of the extracellular matrix and the decreased metabolic activity of the bacteria (Hengzhuang et al. 2011, Zhao et al. 2013, Wu et al. 2015). Biofilms can disperse resistant bacteria into the surrounding environment, resulting in chronic infections (Wu et al. 2015). Thus, non-conventional treatment options are needed, and many systems are currently under development (Kadam et al. 2019). **Hydrogels** have been promoted as wound dressings as they are flexible, biocompatible, and contain water (Caló and Khutoryanskiy 2015, Ullah et al. 2015, Koehler et al. 2018, Smith et al. 2020, Tavakoli and Klar 2020, Brumberg et al. 2021). A multitude of different types of polymers and their combinations can alter the hydrogel properties and be loaded with antimicrobial agents such as gold and silver nanoparticles (Jaiswal et al. 2016, Arafa et al. 2018, Davis et al. 2019), AMPs (Khan et al. 2019), or other synthetic antimicrobial compounds (Nnamani et al. 2014, Aksu et al. 2019). Covering a wound with a hydrogel protects the area while at the same time absorbing wound extrude. The dressing maintains moisture and gradually releases the loaded drugs over longer periods. Polyethylene glycol (PEG) is a widely used polymer, i.e. used for nanoparticles, nanofibers, or fibrin-based wound dressings (Seetharaman et al. 2011, Dubey and Gopinath 2016, Gil et al. 2017). Poloxamers is another polymer that forms thermosreversible hydrogels, which are liquid at lower temperatures and gel with increasing temperatures (Arafa et al. 2018). This feature allows the hydrogel to spread over irregular surfaces, facilitating better wound coverage. In addition to hydrogels that are loaded with antimicrobials, hydrogels with inherent antimicrobial properties have been developed and include naturally occurring substances like peptides or chitosan (Yang et al. 2018). Due to the positive charge of chitosan, it forms ionic interactions with the bacterial cell wall and interferes with its barrier function. This can result in increased susceptibility of the bacteria towards antimicrobial agents (Helander et al. 2001, Ahmadi et al. 2015, Hong et al. 2024). An advantage of these long-release dressings is a potential reduction in dressing changes, promoting less waste, and greater patient compliance. Producing these dressings from sustainably sourced and biodegradable substances will further enhance the sustainability of these approaches.

**Nanofibers and nanoparticles** are potential delivery systems that are well-suited for transport of antimicrobials into skin, because of their size and penetration (Choi and Han 2018). Materials used include a wide range, from common polymers to metals or metal oxides (Altinbasak et al. 2018, Yuan et al. 2018, Hasan et al. 2019). Nanofibers with a nanometer-scale diameter have a high surface-to-volume ratio that, together with their porosity, increase drug load, transport, and cell attachment. It also helps in absorbing wound exudate, nutrient exchange, and protects the area from bacterial attachment (Sill and von Recum 2008, Miguel et al. 2019). Combined mechanisms for drug release, such as near-infrared light, can increase the treatment times by inducing a slower release (Altinbasak et al. 2018). The use of nanofibers in the clinical setting is still, however, limited owing to challenges with toxicity, large-scale production, and functionality (Shahriar et al. 2019). Nanoparticles have garnered increasing interest in the field as they can penetrate deeper tissue layers and affect bacterial growth. The precise design and the huge variety of formulations can elevate antibiotic effects by delivery of high concentrations to the site of infection and a targeted release, reducing side effects (Choi and Han 2018, Altammar 2023, Yusuf et al. 2023). Metallic nanoparticles such as gold, silver, zinc, or copper showed good antimicrobial effects, but care must be taken when it comes to toxic effects in the body (Hemeg 2017, Paduraru et al. 2019, Tao et al. 2019). Silver and other inorganic nanoparticles can also be used to increase the stability of AMPs such as LL-37 (Tsikourkitoudi et al. 2022). Factors including size, surface chemistry, and dissolution are important parameters to optimize antimicrobial activity while reducing toxicity (Smith et al. 2020). Polymeric nanoparticles can be formed for increased biocompatibility and long-term drug release (Hemeg 2017). Emerging technology is also allowing the combination of both antimicrobials and immune modulating factors into the same nanoparticle, which has shown efficacy *in vivo* against different *S. aureus* infections (Lang and Melican 2024, Zhu et al. 2024). Studies have shown the successful use of nanoparticles against a variety of organisms when used on their own or in combination with other systems (Hasan et al. 2019). **Microneedle patches (MNPs)** have been developed for efficient and easy delivery of substances by an array of microneedles with sharp tips. For skin transdermal delivery systems, the microneedles puncture the stratum corneum, the outer skin layer, and reach the underlying skin tissues, which correlates to the location of a number of problematic skin infections (Table 1) (Prausnitz 2017, Lee and Prausnitz 2018, Ziesmer et al. 2021, Nazary Abrbekoh et al. 2022). Depending on strategy, the drug can be loaded into the needle tips, or coated on the surface of the microneedles. The size of the tips as well as the materials used and the MNP design define the depth and mode of drug release. Beside the relatively uncontrolled release in classical MNPs, controllable smart MNPs include mechanisms to modulate the rate, amount, and time of transdermal drug release (Zhang et al. 2016, Raza et al. 2019, Dreiss 2020). Endogenous stimuli such as pH and temperature changes or enzyme-substrate interactions, as well as exogenous stimuli like ultrasounds, UV, NIR, electricity, or electromagnetism, can trigger the release of loaded molecules (Nazary Abrbekoh et al. 2022, Ziesmer et al. 2022, 2023). All these options allow the development and design of advanced MNPs and promising therapeutic approaches. Other potential sustainable approaches that are under development for wound dressings but are not discussed in this review include **natural components**, such as cellulose, aloe, natural plant extracts, and fish skin collagen (Ramanauskienė et al. 2015, Sahiner et al. 2016, Gomaa et al. 2017, Boonmak et al. 2018, Xi et al. 2018, Lukáč et al. 2019). We recognize that to facilitate a new generation of sustainable antimicrobial approaches for treating skin infections, delivery is almost as important as the target. Finding a delivery mechanism that not only delivers high concentration and effective treatment to the right place, but does so in a long-term, sustainable way, is critical. Integrating these treatment and delivery options into existing medical devices is also of interest. A number of these treatment targets as well as delivery mechanisms may be able to be integrated into surface coatings for medical implants or catheters. Biocompatible, local, directed delivery would help address the issues of implant infection, leading to reduced infection, reduced need to replace, and increased sustainability. The interaction of microbiology and immunology with the material science fields and their work with biopolymers and biocompatible delivery systems will help ensure sustainable development of these new treatments.

## Model and study design

The discussed findings and studies highlight the importance of studying host–pathogen interactions and basic research to unravel potentially new targets, approaches, and strategies against SSTIs. However, many questions in this field remain open. A major limitation in basic infection biology has been the reliance on mouse models to study skin infection (Miller et al. 2007, Cho et al. 2010, Kim et al. 2014). The thick, stratified epithelium of human skin presents a vastly different microenvironment compared to rodent skin with different structural and visco-elastic mechanical properties (Wei et al. 2017). The translation of potential therapeutic agents into the human system, the most clinically relevant microenvironment, often fails. It has become increasingly evident that there are significant differences in *Staphylococcus* pathogenesis in human vs mouse systems. Beside structural differences, immunological functions also vary dramatically between these two host environments (Pasparakis et al. 2014). *Staphylococcus* itself demonstrates species-specific virulence, e.g. the species-specific interaction between *Staphylococcus* and langerin, the receptor of skin-resident Langerhans cells (van Dalen et al. 2019). Certain toxins secreted by *S. aureus* act differently against human vs rodent neutrophils (Spaan et al. 2013). With further progress in methodology, **advanced human skin models** are becoming more popular. These models allow experimentation at different complexities, from specific cell types to full tissue systems. Some of the models available include xenografted human skin mouse models, *ex vivo* cultured human skin explants, and 3D cell culture models (Parker 2017, Schulz et al. 2019). The application of these types of human models will become increasingly important as we move towards evaluating new therapeutic approaches, both from the host–pathogen aspect but also in developing delivery methods.

## Conclusion

Treatment of SSTIs has become an increasing challenge in the clinical setting due to rise of multi-drug resistance, biofilm formation, and a complicated wound environment. Together with the increasing awareness of the beneficial microbiota, new treatment options are needed to prevent a commensal microbiome collapse. Despite all the research that has been performed, our knowledge of bacteria-bacteria and host-bacteria interactions in relation to commensals vs pathobionts and skin homeostasis is still limited, particularly in the human microenvironment. Gaining more knowledge in this field together with sophisticated ideas and implementation of nanotechnologies are promising directions for future treatment strategies. The complex composition and environment of human skin raise the **need for interdisciplinary research**, including material sciences, medical biology, microbiology, immunology, and pharmacology. Highlighted in this review, there are a variety of possible novel treatment strategies and as these challenges continue to grow, it is essential to further explore novel treatment and delivery systems to fight skin infections and diseases.

## Data Availability

No new data were generated or analyzed in support of this research.
